# Low estrogen doses normalize testosterone and estradiol levels to the female range in transgender women

**DOI:** 10.6061/clinics/2018/e86

**Published:** 2018-04-21

**Authors:** Flávia Siqueira Cunha, Sorahia Domenice, Maria Helena Palma Sircili, Berenice Bilharinho de Mendonca, Elaine Maria Frade Costa

**Affiliations:** Unidade de Endocrinologia do Desenvolvimento, Laboratorio de Hormonios e Genetica Molecular LIM42, Disciplina de Endocrinologia, Hospital das Clinicas HCFMUSP, Faculdade de Medicina, Universidade de Sao Paulo, Sao Paulo, SP, BR

**Keywords:** Transgender Woman, Male-to-Female Transsexual, Estrogen, Cross-Sex Hormone

## Abstract

**OBJECTIVE:**

The ideal dosage of cross-sex hormones remains unknown. The aim of this study was to evaluate the luteinizing hormone, follicle-stimulating hormone, testosterone, estradiol and prolactin levels after low-dose estrogen therapy with or without cyproterone acetate in transgender women.

**METHODS:**

The serum hormone and biochemical profiles of 51 transgender women were evaluated before gonadectomy. Hormone therapy consisted of conjugated equine estrogen alone or combined with cyproterone acetate. The daily dose of conjugated equine estrogen was 0.625 mg in 41 subjects and 1.25 mg in 10 subjects, and the daily dose of cyproterone acetate was 50 mg in 42 subjects and 100 mg in one subject.

**RESULTS:**

Estrogen-only therapy reduced the testosterone, luteinizing hormone and follicle-stimulating hormone levels from 731.5 to 18 ng/dL, 6.3 to 1.1 U/L and 9.6 to 1.5 U/L, respectively. Estrogen plus cyproterone acetate reduced the testosterone, luteinizing hormone and follicle-stimulating hormone levels from 750 to 21 ng/dL, 6.8 to 0.6 U/L and 10 to 1.0 U/L, respectively. The serum levels of luteinizing hormone, follicle-stimulating hormone, testosterone, estradiol and prolactin in the patients treated with estrogen alone and estrogen plus cyproterone acetate were not significantly different. The group receiving estrogen plus cyproterone acetate had significantly higher levels of gamma-glutamyltransferase than the group receiving estrogen alone. No significant differences in the other biochemical parameters were evident between the patients receiving estrogen alone and estrogen plus cyproterone acetate.

**CONCLUSION:**

In our sample of transgender women, lower estrogen doses than those usually prescribed for these subjects were able to adjust the testosterone and estradiol levels to the physiological female range, thus avoiding high estrogen doses and their multiple associated side effects.

## INTRODUCTION

Transgender women (TW) have a female gender identity and desire to live as a member of the female community 1. Cross-sex identification typically has an early onset and causes chronic suffering. To alleviate the distress associated with belonging to an undesired gender group, sex reassignment hormone therapy is indicated and must be preceded by a thorough psychological evaluation to establish the diagnosis of gender dysphoria 2.

The therapeutic process for TW includes three mainstays: psychotherapy, hormone therapy and sex reassignment surgery 3. During the process of changing from the male to the female phenotype, TW require the administration of cross-sex hormones to mitigate the phenotypic signs of the male biological sex and to develop female characteristics. After hormone therapy, expected physical changes include breast development and the redistribution of body fat to a female pattern, as well as a decrease in facial hair growth, body hair growth, muscle mass, testicular volume and spontaneous erections 4.

The aim of hormone therapy in TW is to promote the development of female phenotypic characteristics using the lowest effective estrogen doses to maintain serum estradiol (E2) and testosterone (T) levels within the normal range for women in the follicular phase of the menstrual cycle 3,5.

The treatment regimen of TW usually consists of estrogen combined with a compound that suppresses androgen actions, such as spironolactone, finasteride, flutamide or cyproterone acetate (CA) 3. Spironolactone has a synergistic effect with estrogen on physical changes 6. Finasteride and flutamide are rarely used; the efficacy of finasteride is limited, and flutamide is associated with liver toxicity 7.

CA is the most commonly used antiandrogen drug in Europe and South America. CA acts as a potent competitive antagonist of the androgen receptor and has additional progestational activity, inhibiting luteinizing hormone (LH) release 8.

Different estrogen compounds and routes of administration exist for cross-sex hormone treatment. The use of oral ethinyl E2 in transsexuals is associated with an increased risk of venous thromboembolism and death from cardiovascular events 9,10. Transdermal preparations are the safest forms of estrogen administration, but oral 17β-E2, conjugated equine estrogens (CEEs) and E2 valerate are also safer options than oral ethinyl E2 for use in cross-sex hormone treatment 3,11-13. The recommended estrogenic doses for feminization of TW, according to the Endocrine Society guidelines, are usually three times higher than those used for hormone replacement therapy in postmenopausal women and are similar to those used in hypogonadal patients 3,14.

We retrospectively analyzed the effects of lower doses of estrogen in TW on suppressing endogenous T and maintaining the physiological levels of E2 within the normal range for premenopausal women in the follicular phase.

## MATERIALS AND METHODS

This retrospective register study was approved by the Ethics Committee of the Hospital das Clínicas da Faculdade de Medicina da Universidade de São Paulo (HCFMUSP), and signed informed consent forms were obtained from all patients.

All patients consulting in our Transsexual Unit with biochemical and hormonal data before and 6 months after therapy were invited to participate in this retrospective analysis, and of the 58 patients who were invited, 51 agreed to participate. The patients had a confirmed diagnosis of transsexualism/gender dysphoria based on the International Classification of Diseases (ICD-10) and Diagnostic and Statistical Manual of Mental Disorders (DSM-V) criteria and were assessed by mental health professional experts in gender dysphoria 15,16.

The mean age at the first evaluation was 38.3±7.4 years (range 23-58 years). All patients had a normal male phenotype (46, XY chromosome karyotype), and none of them had previously undergone an orchiectomy.

The hormonal profiles (LH, follicle-stimulating hormone [FSH], T, E2 and prolactin [PRL] levels) and biochemical analyses (glycemia, total cholesterol [TC], high-density lipoprotein [HDLc], low-density lipoprotein [LDLc], very low-density lipoprotein [VLDLc], triglycerides [TG], aspartate transaminase [AST], alanine transaminase [ALT] and gamma-glutamyltransferase [GGT] levels) were evaluated at the baseline condition, after the withdrawal of all hormone therapy for at least 3 months, and after 6 months of supervised hormone therapy.

Cross-sex hormone therapy consisted of oral CEE alone and oral CEE combined with CA in 8 and 43 patients, respectively. The daily doses of estrogen were 0.625 mg (41 subjects) or 1.25 mg (10 subjects), and the daily doses of CA were 50 mg (42 subjects) and 100 mg (1 subject).

### Hormone assays

The LH, FSH, T, E2 and PRL levels were measured with immunofluorometric assays (AutoDELFIA, Wallac, Finland). The intra- and inter-assay coefficients of variation varied from 5% to 10%.

### Biochemical analysis

The fasting glucose levels were determined with an automatic enzymatic colorimetric method using hexokinase (Cobas Integra; Roche, Basel, Switzerland). The TC, HDLc, LDLc, VLDLc and TG levels were analyzed with an automatic enzymatic colorimetric method (Cobas Mira; F. Hoffmann-La Roche, Basel, Switzerland). AST, ALT and GGT were measured with an automated biochemistry analyzer (Roche, Mannheim, Germany).

### Statistical analysis

The nonparametric Wilcoxon test was used to compare the hormone levels before and after treatment. The nonparametric Mann-Whitney test was used to compare the different treatments. For continuous variables with normal distributions, Student’s t-test was applied. A significance level of 5% was adopted in this study. The software used was STATA version 13.0 (StataCorp LP, College Station, Texas, USA).

## RESULTS

Cross-sex hormone treatment with estrogen alone (n=8 patients) reduced the T levels from 731.5 to 18 ng/dL (*p*=0.01). The LH and FSH levels were also reduced from 6.3 to 1.1 U/L (*p*=0.049) and 9.6 to 1.5 U/L (*p*=0.01), respectively (Table 1). In this group of patients, four subjects received 0.625 mg CEE daily, and four received 1.25 mg CEE daily; no differences were evident between the hormone levels in these patient subgroups (Table 2).

Estrogen plus CA therapy (n=43 patients) reduced the T levels from 750 to 21 ng/dL (*p*<0.001). The LH and FSH levels were also reduced from 6.8 to 0.6 U/L (*p*<0.001) and 10 to 1.0 U/L (*p*<0.001), respectively (Table 1). Thirty-seven subjects received 0.625 mg CEE daily, and 6 subjects received 1.25 mg daily; all subjects received 50 mg CA (42 subjects) or 100 mg CA (1 subject) daily. After comparing these two CEE subgroups, we observed that both treatments were able to suppress the T levels to within the normal female range (22 *vs* 18.9 ng/dL, *p*=0.3) (Table 2).

The estrogen levels before and after cross-sex hormone treatment were not significantly different (*p*=0.6) (Table 1). In contrast, the PRL levels increased after low-dose cross-sex hormone therapy (*p*=0.004) (Table 1), with statistical significance only in the ‘estrogen plus CA’ group (*p*=0.009; data not shown). Although an increase in the PRL levels was observed in some patients after hormone therapy, only four individuals had PRL values higher than the normal range (2-15 ng/mL). The values were 48.9, 36 and 36.7 ng/mL for three patients receiving 0.625 mg CEE plus 50 mg CA daily and 30 ng/mL for one patient receiving 0.625 mg CEE daily.

The serum levels of LH, FSH, T, E2 and PRL in the patients treated with estrogen alone or estrogen plus CA for 6 months were not significantly different (*p*>0.05) (Table 3).

No significant differences were evident for the biochemical levels of glycemia (86±6 *vs* 86±13, *p*=0.8), TC (170±37 *vs* 173±34, *p*=0.7), HDLc (49±10 *vs* 50±14, *p*=0.7), LDLc (107±36 *vs* 106±32, *p*=0.9), VLDLc (20±14 *vs* 19±12, *p*=0.8), TG (84±34 *vs* 87±39, *p*=0.7), AST (22±7 *vs* 20±6, *p*=0.4), ALT (20±9 *vs* 19±9, *p*=0.9) and GGT (24±14 *vs* 26±13, *p*=0.6) before *vs* after cross-sex hormone therapy, respectively.

The group receiving estrogen plus CA presented higher levels of GGT than the group receiving estrogen alone (16±7 *vs* 24±7, *p*=0.009). No significant differences were evident for glycemia (98±22 *vs* 83±7, *p*=0.1), TC (168±40 *vs* 161±17, *p*=0.7), HDLc (54±11 *vs* 53±13, *p*=0.3), LDLc (94±36 *vs* 94±16, *p*=0.4), VLDLc (19±13 *vs* 14±5, *p*=0.9), TG (96±64 *vs* 76±19, *p*=0.7), AST (23±8 *vs* 18±3, *p*=0.3) and ALT (19±12 *vs* 19±11, *p*=0.9) in the ‘estrogen-alone group’ *vs* the ‘estrogen-plus-CA group’, respectively.

## DISCUSSION

TW generally have a psychological need to increase estrogen replacement doses to acquire a female phenotype as soon as possible. A higher estrogen dose is associated with an increased risk of venous thromboembolic disease, pulmonary embolism, myocardial infarction, stroke, hormone-related tumors and adverse liver effects 17. In addition to these common side effects of high-dose estrogen therapy, increases in PRL levels and even prolactinoma development have been described in TW 18-24.

The ideal dosage of cross-sex hormones is still unknown because randomized controlled trials in this specific transgender population are not available. The multiple types of estrogens, variability of their measurement and different routes of administration make the standardization of therapeutic regimens difficult. Long-term follow-up studies of hormone treatment in transsexuals and of hormone replacement therapy in biological females are used to guide cross-sex hormone therapy in TW 3.

The use of a synthetic estrogen, ethinyl E2, in a large cohort of transsexuals has been associated with an increased risk of cardiovascular and thromboembolic events. Interestingly, the vast majority of these adverse events occur during the first year of estrogen treatment, and the risk is higher in patients older than 40 years 9,10. Natural estrogens are safer options than synthetic estrogens for cross-sex hormone treatment 11-13. Transdermal preparation is the safest form of estrogen administration, especially in transsexual patients who smoke or have diabetes because the transdermal preparation does not influence protein, lipoprotein or triglyceride synthesis, thereby reducing the thrombotic and cardiovascular risks 3.

In our hospital, transsexual patients are treated by a multidisciplinary group and receive psychological support before and after surgery. The psychological support controls the anxiety of the patients as they develop the female phenotype and allows us to treat our patients with the lowest estrogen doses necessary to normalize androgen and estrogen levels.

This study is the first to evaluate the use of low doses of estrogens in TW. We have demonstrated that low estrogen doses alone or with CA are effective toward maintaining androgen suppression and serum E2 within the normal follicular-phase range.

Evaluations of the effects of low-dose estrogen therapy on physical changes, namely, breast development, facial hair growth, body hair growth and body fat redistribution, were not possible in our patient population because all of the patients reported prior use of other estrogen formulations without medical supervision for a variable period of time. However, the maintenance of estrogen levels in the normal female range suggests that low-dose estrogen therapy may be able to promote the satisfactory feminization of these patients. The facial hair response to hormonal treatment in transsexuals, even at high estrogen doses, is very poor and often requires complementary cosmetic treatments such as laser treatments and electrolysis. In addition, many signs of feminization, including breast development, depend on not only the estrogen dose but also the individual patient’s sensitivity to estrogen. In a cohort of transsexuals receiving high doses of estrogen, the result of breast augmentation from hormones was described as modest, and the rate of breast augmentation surgery in a series of transsexuals was generally as high as 70% 25-27. Nevertheless, all patients in our cohort achieved an advanced Tanner’ stage (IV and V) in breast development.

The use of CA is especially important to alleviate androgenic signs and symptoms such as the male pattern of facial and body hair and the undesired spontaneous penile erections frequently reported by TW. In our analysis, we noted that CA was not critical for achieving androgen suppression and that estrogen alone, even at low doses, was effective toward suppressing the hypothalamic-pituitary-testicular axis 8,28. Even so, we observed that the regimen comprising estrogen plus CA achieved a more potent suppression of LH levels than the regimen containing estrogen alone (from 6.8 to 0.6 IU/L, *p*<0.001 *vs* from 6.3 to 1.1 IU/L, *p*=0.049, respectively).

In our clinical practice, we avoid daily doses higher than 50 mg of CA because of its potential metabolic side effects, including weight gain and high blood pressure. Moreover, ethinyl E2 plus CA might induce a degree of insulin resistance in TW 29. We observed that the GGT levels were higher in the group receiving estrogen plus CA than in the group receiving estrogen alone, although the both groups presented with GGT levels in the normal range.

In our cohort, the estrogen levels before and after cross-sex hormone treatment were not significantly different. The conversion of male levels of T to estrogen before treatment was similar to that achieved by the low-dose estrogen treatment 30.

Estrogen-induced increases in PRL levels in many physiological conditions, such as pregnancy and puberty, are well known, and a mild increase in PRL levels in TW has been described after estrogen therapy 31. Similarly, higher PRL levels were identified in our cohort after low doses of estrogen therapy. Although these levels were not statistically significant in the group that received estrogen alone, a significant increase was observed in the group receiving estrogen plus CA. Other authors have reported increased PRL levels associated with CA 32-34.

Our results demonstrated that the different doses of CEE (0.625 *vs* 1.25 mg daily) were not significant in terms of the elevation in the PRL level, probably due to the low dose of estrogen in both subgroups. Additionally, we observed that the two different doses of CEE had similar effects on hormone levels after 6 months of treatment. However, these data should be regarded with caution because the number of individuals in each subgroup is very small, thus reducing the statistical power of the sample.

In conclusion, in our sample of TW, lower estrogen doses than those usually prescribed for these subjects were able to adjust the T and E2 levels to the physiological female range, avoiding the risks of high estrogen doses. Both regimens, namely, CEE alone or with CA, achieved the laboratory goals in the treatment of TW.

## AUTHOR CONTRIBUTIONS

Mendonça BB, Costa EM and Domenice S were responsible for the study conception and design and critical revision of the manuscript. Cunha FS and Sircili MH were responsible for the acquisition and analysis of data, and manuscript drafting.

## Figures and Tables

**Table 1:** Serum hormone levels of 51 transgender women before and after 6 months of low-dose estrogen therapy with or without cyproterone acetate.

	Baseline (no CSH)			Six months (on CSH)			p value*
	Estrogen alone	Estrogen plus CA	Median baseline*	Estrogen alone	Estrogen plus CA	Median 6 months*	
	Median (variation)	Median (variation)		Median (variation)	Median (variation)		
LH (U/L)	6.3 (3.0-10.6)	6.8 (3.4-26.5)	6.6 (3.0-26.5)	1.1 (0.1-7.7)	0.6 (0.1-2.5)	0.6 (0.1-7.7)	<0.001
FSH (U/L)	9.6 (1.5-18.2)	10.0 (1.5-36.5)	10.0 (1.5-36.5)	1.5 (1.0-7.7)	1.0 (0.7-7.7)	1.0 (0.7-7.7)	<0.001
Testosterone (ng/dL)	731.5 (495-935)	750.0 (228-1420)	750 (228-1420)	18.0 (11-21)	21.0 (11-72)	20 (11-72)	<0.001
Estradiol (pg/mL)	45.3 (13.0-53.9)	36.0 (19.9-92.0)	36.6 (13.0-92.0)	38.2 (13.0-61.0)	34.0 (13.0-88.0)	36.0 (13.0-88.0)	0.649
Prolactin (ng/mL)	7.6 (3.9-17.9)	7.6 (3.9-17.9)	7.6 (3.9-17.9)	16.3 (3.4-48.9)	16.3 (8.0-48.9)	16.3 (3.4-48.9)	0.004

Normal value for premenopausal women at the follicular phase of the menstrual cycle: LH, 2.2-6.8 IU/L; FSH, 2.4-9.3 IU/L; Testosterone, <98 ng/dL; Estradiol, 22-215 pg/mL; Prolactin, 2-15 ng/mL.

CA: cyproterone acetate; CSH: cross-sex hormone.

* Median baseline compared to the median at 6 months.

**Table 2:** Serum hormone levels after 6 months of treatment with different doses of conjugated equine estrogen alone or with cyproterone acetate in transgender women.

	0.625 mg CEE (n=4)*	1.25 mg CEE (n=4)*	p value*	0.625 mg CEE plus 50 mg VA (n=37)**	1.25 mg CEE plus 50 mg CA (n=6)**	p value**
	Median (variation)	Median (variation)		Median (variation)	Median (variation)	
LH (U/L)	1.1 (0.6-6.9)	0.8 (0.1-7.7)	0.561	0.6 (0.1-2.5)	0.7 (0.1-0.9)	0.619
FSH (U/L)	1.9 (1.0-7.7)	1.5 (0.1-2.1)	0.539	1.0 (0.7-7.7)	1.0 (1.0-3.3)	0.732
Testosterone (ng/dL)	15.5 (11-20)	19.5 (16-21)	0.191	22.0 (11-72)	18.9 (11-28)	0.292
Estradiol (pg/mL)	38.2 (13.0-45.1)	39.3 (13.0-61.0)	0.633	34.0 (13.0-88.0)	33.3 (18.0-68.0)	0.874
Prolactin (ng/mL)	5.2 (3.1-7.3)	12.0 (3.4-36.7)	0.355	15.5 (8.0-48.9)	14.7 (12.8-19.1)	0.874

Normal value for premenopausal women at the follicular phase of the menstrual cycle: LH, 2.2-6.8 IU/L; FSH, 2.4-9.3 IU/L; Testosterone, <98 ng/dL; 17β-estradiol, 22-215 pg/mL; Prolactin, 2-15 ng/mL.

CEE: conjugated equine estrogen; CA: cyproterone acetate.

* 0.625 mg CEE compared to 1.25 mg CEE.

** 0.625 mg CEE plus 50 mg CA compared to 1.25 mg CEE plus 50 mg CA.

**Table 3:** Serum hormone levels in transgender women after 6 months of conjugated equine estrogen therapy with or without cyproterone acetate therapy.

	CEE plus CA (n=43)	CEE alone (n=8)	*p value*
	Median (variation)	Median (variation)	
LH (U/L)	0.6 (0.1-2.5)	1.1 (0.1-7.7)	0.313
FSH (U/L)	1.0 (0.7-7.0)	1.5 (1.0-7.7)	0.594
Testosterone (ng/dL)	21.0 (11-72)	18.0 (11-21)	0.217
Estradiol (pg/mL)	34.0 (13.0-88.0)	38.2 (13.0-61.0)	0.835
Prolactin (ng/mL)	15.5 (8.0-48.9)	5.8 (3.1-36.7)	0.126

Normal value for premenopausal women at the follicular phase of the menstrual cycle: LH, 2.2-6.8 IU/L; FSH, 2.4-9.3 IU/L; Testosterone, <98 ng/dL; 17β-estradiol, 22-215 pg/mL; Prolactin, 2-15 ng/mL.

CEE: conjugated equine estrogen; CA: cyproterone acetate.

## References

[1] Cohen-Kettenis PT, Gooren LJ (1999). Transsexualism: a review of etiology, diagnosis and treatment. J Psychosom Res.

[2] Cohen-Kettenis PT, Pfäfflin F (2010). The DSM diagnostic criteria for gender identity disorder in adolescents and adults. Arch Sex Behav.

[3] Hembree WC, Cohen-Kettenis P, Delemarre-van de Waal HA, Gooren LJ, Meyer WJ, Spack NP (2009). Endocrine treatment of transsexual persons: an Endocrine Society clinical practice guideline. J Clin Endocrinol Metab.

[4] Moore E, Wisniewski A, Dobs A (2003). Endocrine treatment of transsexual people: a review of treatment regimens, outcomes, and adverse effects. J Clin Endocrinol Metab.

[5] Gooren LJ (2011). Clinical practice. Care of transsexual persons. N Engl J Med.

[6] Prior JC, Vigna YM, Watson D (1989). Spironolactone with physiological female steroids for presurgical therapy of male-to-female transsexualism. Arch Sex Behav.

[7] Knezevich EL, Viereck LK, Drincic AT (2012). Medical management of adult transsexual persons. Pharmacotherapy.

[8] Gooren LJ, Giltay EJ, Bunck MC (2008). Long-term treatment of transsexuals with cross-sex hormones: extensive personal experience. J Clin Endocrinol Metab.

[9] Asscheman H, Gooren LJ, Eklund PL (1989). Mortality and morbidity in transsexual patients with cross-gender hormone treatment. Metabolism.

[10] van Kesteren PJ, Asscheman H, Megens JA, Gooren LJ (1997). Mortality and morbidity in transsexual subjects treated with cross-sex hormones. Clin Endocrinol.

[11] Asscheman H, Giltay EJ, Megens JA, de Ronde WP, van Trotsenburg MA, Gooren LJ (2011). A long-term follow-up study of mortality in transsexuals receiving treatment with cross-sex hormones. Eur J Endocrinol.

[12] Gooren LJ, Wierckx K, Giltay EJ (2014). Cardiovascular disease in transsexual persons treated with cross-sex hormones: reversal of the traditional sex difference in cardiovascular disease pattern. Eur J Endocrinol.

[13] Asscheman H, T′Sjoen G, Lemaire A, Mas M, Meriggiola MC, Mueller A (2014). Venous thrombo-embolism as a complication of cross-sex hormone treatment of male-to-female transsexual subjects: a review. Andrologia.

[14] Moreno-Pérez O, Esteva De Antonio I (2012). Clinical practice guidelines for assessment and treatment of transsexualism. SEEN Identity and Sexual Differentiation Group (GIDSEEN). Endocrinol Nutr.

[15] Drescher J (2015). Queer diagnoses revisited: The past and future of homosexuality and gender diagnoses in DSM and ICD. Int Rev Psychiatry.

[16] Lawrence AA (2014). Gender assignment dysphoria in the DSM-5. Arch Sex Behav.

[17] Levy A, Crown A, Reid R (2003). Endocrine intervention for transsexuals. Clin Endocrinol.

[18] Gooren LJ, Assies J, Asscheman H, de Slegte R, van Kessel H (1988). Estrogen-induced prolactinoma in a man. J Clin Endocrinol Metab.

[19] Kovacs K, Stefaneanu L, Ezzat S, Smyth HS (1994). Prolactin-producing pituitary adenoma in a male-to-female transsexual patient with protracted estrogen administration. A morphologic study. Arch Pathol Lab Med.

[20] Serri O, Noiseux D, Robert F, Hardy J (1996). Lactotroph hyperplasia in an estrogen treated male-to-female transsexual patient. J Clin Endocrinol Metab.

[21] Mueller A, Gooren L (2008). Hormone-related tumors in transsexuals receiving treatment with cross-sex hormones. Eur J Endocrinol.

[22] García-Malpartida K, Martín-Gorgojo A, Rocha M, Gómez-Balaguer M, Hernández-Mijares A (2010). Prolactinoma induced by estrogen and cyproterone acetate in a male-to-female transsexual. Fertil Steril.

[23] Wierckx K, Elaut E, Declercq E, Heylens G, De Cuypere G, Taes Y (2013). Prevalence of cardiovascular disease and cancer during cross-sex hormone therapy in a large cohort of trans persons: a case-control study. Eur J Endocrinol.

[24] Cunha FS, Domenice S, Câmara VL, Sircili MH, Gooren LJ, Mendonça BB (2015). Diagnosis of prolactinoma in two male-to-female transsexual subjects following high-dose cross-sex hormone therapy. Andrologia.

[25] Orentreich N, Durr NP (1974). Proceedings: Mammogenesis in transsexuals. J Invest Dermatol.

[26] Kanhai RC, Hage JJ, Karim RB (2001). Augmentation mammaplasty in male-to-female trans-sexuals: facts and figures from Amsterdam. Scand J Plast Reconstr Surg Hand Surg.

[27] Wierckx K, Gooren L, T′Sjoen G (2014). Clinical review: Breast development in trans women receiving cross-sex hormones. J Sex Med.

[28] Weyers S, Elaut E, De Sutter P, Gerris J, T′Sjoen G, Heylens G (2009). Long-term assessment of the physical, mental, and sexual health among transsexual women. J Sex Med.

[29] Elbers JM, Giltay EJ, Teerlink T, Scheffer PG, Asscheman H, Seidell JC (2003). Effects of sex steroids on components of the insulin resistance syndrome in transsexual subjects. Clin Endocrinol.

[30] MacDonald PC, Madden JD, Brenner PF, Wilson JD, Siiteri PK (1979). Origin of estrogen in normal men and in women with testicular feminization. J Clin Endocrinol Metab.

[31] Asscheman H, Gooren LJ, Assies J, Smits JP, de Slegte R (1988). Prolactin levels and pituitary enlargement in hormone-treated male-to-female transsexuals. Clin Endocrinol.

[32] Fonzo D, Angeli A, Sivieri R, Andriolo S, Frajria R, Ceresa F (1977). Hyperprolactinemia in girls with idiopathic precocious puberty under prolonged treatment with cyproterone acetate. J Clin Endocrinol Metab.

[33] Willemse PH, Dikkeschei LD, Mulder NH, van der Ploeg E, Sleijfer DT, de Vries EG (1988). Clinical and endocrine effects of cyproterone acetate in postmenopausal patients with advanced breast cancer. Eur J Cancer Clin Oncol.

[34] Paoletti AM, Floris S, Mannias M, Orrù M, Crippa D, Orlandi R (2001). Evidence that cyproterone acetate improves psychological symptoms and enhances the activity of the dopaminergic system in postmenopause. J Clin Endocrinol Metab.

